# The association between maximum mouth opening and sociodemographic factors among Saudi adults: a cross-sectional study

**DOI:** 10.3389/froh.2025.1724913

**Published:** 2026-01-12

**Authors:** Bayan Almohaimeed, Rashad Ikram

**Affiliations:** 1 Department of Community Dentistry and Oral Epidemiology, College of Dentistry, Qassim University, Qassim, Saudi Arabia; 2 Consultant, Department of Oral and Maxillofacial Surgery, Buraidah Central Hospital, Qassim Health Cluster, Qassim, Saudi Arabia

**Keywords:** mandibular mobility, maximum mouth opening (MMO), oral health assessment, sociodemographic factors, temporomandibular joint (TMJ), trismus

## Abstract

**Introduction:**

Maximum mouth opening (MMO) is an essential clinical measurement for evaluating mandibular function and temporomandibular joint mobility. Establishing normative values across populations is crucial for the accurate diagnosis of trismus and for guiding oral and maxillofacial interventions. This study aimed to determine the mean MMO among Saudi adults and to examine its association with sociodemographic and anthropometric variables.

**Methods:**

A cross-sectional study was conducted among 400 adults in Qassim, Saudi Arabia. MMO was measured using a digital caliper with triplicate readings averaged for analysis. Sociodemographic and anthropometric data were recorded. Group differences in MMO were assessed using one-way analysis of variance (ANOVA), followed by Tukey post hoc testing for variables with statistically significant ANOVA results, in addition to Monte Carlo-adjusted chi-square tests for categorical comparisons. A multivariable linear regression model was performed to identify independent predictors of MMO.

**Results:**

The mean of MMO was 45.7 ± 6.9 mm (median: 46.3 mm; range: 28.0–66.3 mm). ANOVA showed significant differences in MMO by gender (*p* < 0.0001) and height (*p* < 0.0001), with males and taller individuals exhibiting greater mouth opening. No significant differences were found across age, weight, or BMI categories. In the multivariable regression model, height remained the only significant independent predictor of MMO (*β* = 0.154, *p* = 0.008), while gender showed a non-significant trend. Age and weight were not associated with MMO.

**Discussion:**

In this adult Saudi population, MMO was primarily influenced by height, with males showing larger values. Age, weight, and BMI were not significant predictors. These findings support using sex and stature-adjusted reference values to improve trismus diagnosis and clinical decision-making. Further research incorporating craniofacial measures is recommended to refine normative standards.

## 1 Introduction

Maximum mouth opening (MMO) is a fundamental clinical measurement used to evaluate mandibular function and temporomandibular joint (TMJ) mobility. It is defined as “the greatest distance between the incisal edge of the maxillary central incisors to the incisal edge of the mandibular central incisors at the midline when the mouth is opened as wide as possible” (1). MMO is commonly measured in millimeters and serves as a critical diagnostic and prognostic indicator in various fields, including dentistry, oral and maxillofacial surgery, otolaryngology, and physiotherapy. Restricted mouth opening, including trismus, can significantly compromise quality of life by limiting essential functions such as chewing, speaking, swallowing, and performing oral hygiene. It also hinders routine dental care and surgical access, contributing to treatment delays and increased morbidity. Psychosocial impacts, including communication difficulty, social discomfort, and reduced well-being, have also been documented (2, 3). These consequences underscore the clinical relevance of establishing accurate normative values for maximum mouth opening.

Normal MMO values vary considerably among individuals and are influenced by factors such as age, sex, ethnicity, and craniofacial morphology (1, 4, 5). Therefore, establishing normative data across diverse populations is important for accurate clinical assessment. Treating patients with limited mouth opening can be challenging for dentists. Reduced MMO may result from a wide range of pathological conditions and contributing factors, including temporomandibular joint (TMJ) disorders, infections, ankylosis, fractures of the mandible and midfacial region, dental impactions, tumors, soft tissue scarring, and exposure to head and neck radiotherapy (5). Conversely, hypermobility may also pose clinical concerns in certain settings.

In Saudi Arabia, a study by Alhammad reported a mean MMO of 47.8 ± 6.9 mm, with a significant association between MMO and height and weight (6). Another study by Al-Dlaigan (7), involving adolescents aged 12–16 years, found the mean maximal mouth opening for males was 43.5 ± 4.23 mm (range 29–59 mm) and for females 35.5 ± 4.4 mm (range 20–45 mm) (7). Moreover, across all age groups, the mean MMO for men was significantly higher than that for women in the study by Fatima et al. (8). Other studies have investigated the relationship between MMO and facial types, gender, height, weight, and body mass index (BMI) (6, 8).

A study among Turkish adults with 1,582 participants reported a mean MMO of 44.2 mm for men and 40.29 mm for women. A significant correlation was observed between MMO and height (*p* < 0.05) (9). For the Jordanian population, Sawair et al. reported the Active Maximum Mouth Opening (AMMO) for men as 45.3 ± 5.7 mm and for women as 41.5 ± 5.3 mm. The AMMO was significantly associated with height and weight (10).

Measurement of maximum mouth opening in normal subjects across different age groups is beneficial for managing patients with maxillofacial injuries and other anomalies. It guides surgeons in restoring mouth opening to levels considered normal. This study has two objectives: first, to assess maximal mouth opening in normal Saudi adults; and second, to examine the relationship between maximum mouth opening (measured across three attempts) and various sociodemographic factors.

## 2 Materials and methods

A cross-sectional study was conducted involving adult patients from the Oral and Maxillofacial dental clinics at the Dental Center, Buraydah Central Hospital. The study received ethical approval from the Regional Research Ethics Committee, Qassim Province, Code #:607/ 45/13562. The identities of the participants were kept confidential throughout the research process. Participants' consent was obtained, and they were informed in advance of the study's rationale and objectives; their participation was voluntary.

Data were collected by (R.I) from July 2024 to July 2025 through interviews to record participants' demographic data and clinical examinations. An electronic digital caliper was used to measure patients' maximum mouth opening in the clinic.

### 2.1 The sample size

A non-probability voluntary sampling technique was used. The required sample size was estimated using OpenEpi (Version 3.01) for a descriptive cross-sectional study. In the absence of prior Saudi-specific estimates of maximum mouth opening (MMO), a conservative expected proportion of 50% was used, as it generates the largest sample size for a specified precision level. The calculation assumed a 95% confidence interval, a 5% margin of error, 80% statistical power, and a design effect of 1.0. Based on these parameters, the minimum required sample size was 385 participants. To enhance the precision of estimates and permit stratified analyses, the final sample included 408 participants.

### 2.2 Inclusion and exclusion criteria

Participants were eligible for inclusion if they were 18 years of age or older and provided informed consent. Individuals younger than 18 years or unwilling to participate were excluded. Additional exclusion criteria included a history of facial trauma, facial tumors, active infections, craniofacial deformities, trismus, missing maxillary or mandibular anterior teeth, or systemic conditions known to affect temporomandibular joint function. To ensure accuracy in caliper placement, no correction factor was applied for missing or uneven incisal edges; therefore, participants with severe incisal wear, fractured incisal edges, pronounced anterior tooth irregularities, or the absence of intact central incisors were excluded from measurement. The digital caliper was consistently positioned on the most superior incisal points of the maxillary and mandibular central incisors. Screening for these criteria was conducted through a combination of: (A) clinical oral examination to identify missing anterior teeth, trismus, or TMJ abnormalities; (B) structured interview questions in which participants self-reported systemic diseases, trauma history, and previous diagnoses; and (C) cross-checking with available electronic hospital records when systemic diseases were reported. A total of eight participants met the exclusion criteria and were removed from the final analysis.

### 2.3 Method for measuring the mouth opening

**Figure 1:**
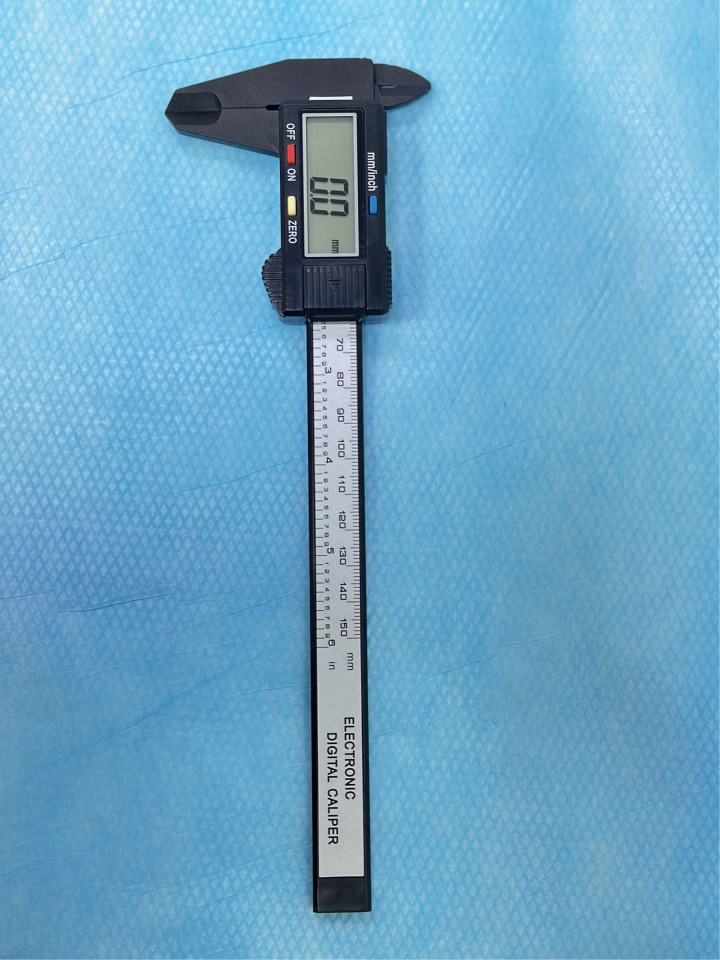
Closed electronic digital caliper.

**Figure 2:**
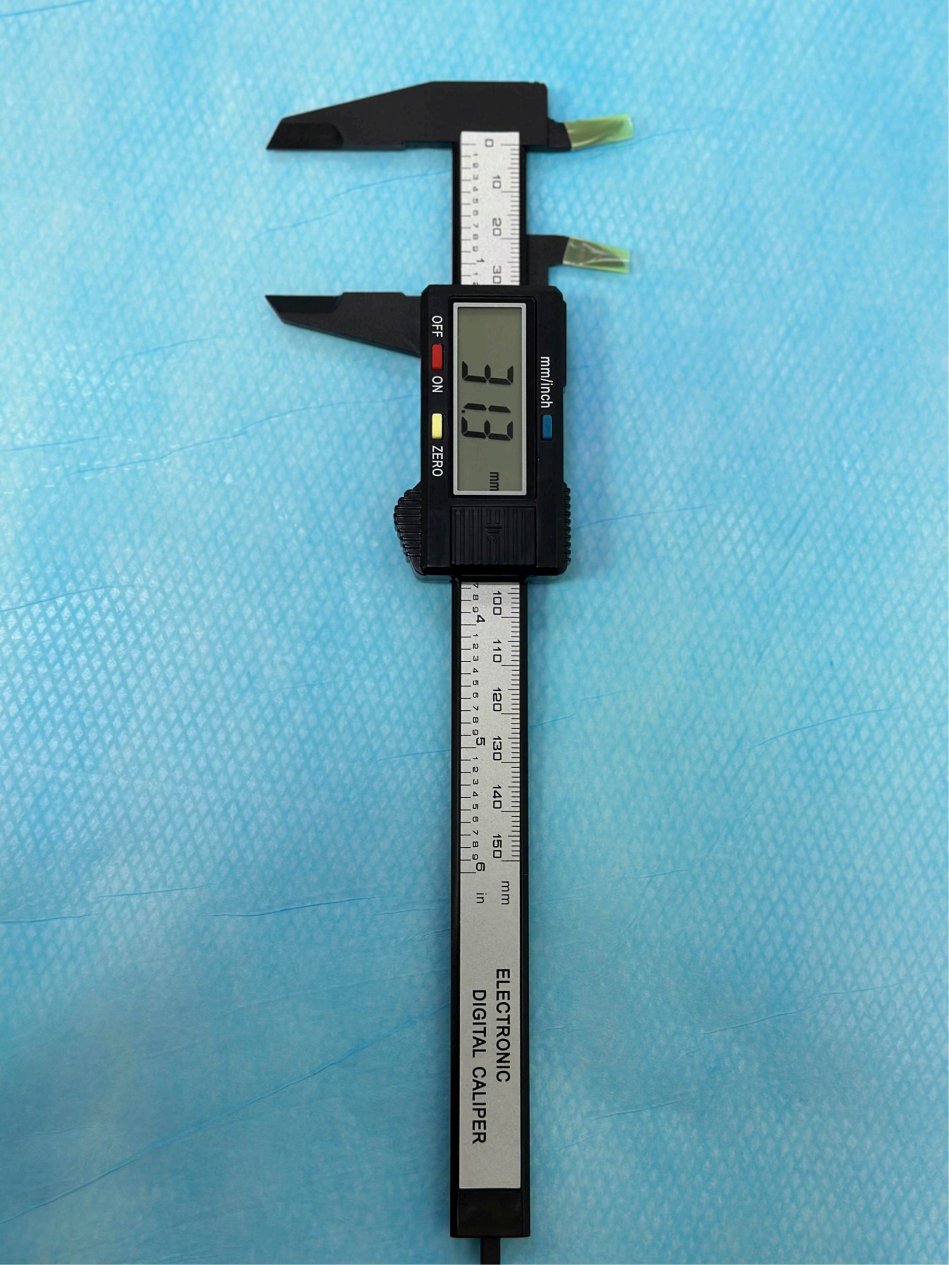
Open electronic digital caliper.

The maximum mouth opening (MMO) was measured using a calibrated digital caliper. For infection control, the tips of the clipper were covered for each patient, and zero calibration was done before starting the process. Participants were seated in an upright position with the head aligned in a natural resting posture, and measurements were taken from a frontal view. Each subject was instructed to open the mouth maximally until no additional movement was possible. Following verification of a zero reading, the calipers inside jaws were carefully positioned between the maxillary and mandibular central incisors and gently expanded until both opposing incisal edges were in contact with the measuring surfaces. The distance was recorded in millimeters to two decimal places. Three consecutive measurements were obtained for each participant, and the mean value was used for analysis. After each measurement, the caliper tips were cleaned by removing the protective cover, disinfecting with ethyl alcohol, and wiping with sterile cotton (Figures 1, 2).

### 2.4 Statistical analysis

Data were analyzed using SAS OnDemand for Academics. Descriptive statistics were computed for all variables, including means, medians, ranges, and standard deviations for continuous variables, and frequencies and percentages for categorical variables.

Associations between maximum mouth opening (MMO) and categorical sociodemographic variables (age groups, gender, height categories, weight categories, and BMI groups) were initially assessed using Pearson's Chi-square test. For contingency tables with expected cell counts <5, the Exact Pearson Chi-square test with Monte Carlo simulation was used. To examine differences in mean MMO across sociodemographic categories, one-way Analysis of Variance (ANOVA) was performed for age, gender, height, weight, and BMI. For variables with statistically significant ANOVA results, Tukey *post hoc* testing was conducted to determine pairwise differences between specific category levels. To identify independent predictors of MMO treated as a continuous outcome, a multivariable linear regression model was constructed, including age, gender, height, and weight as covariates. Model fit was evaluated using *R*^2^, F-statistics, and examination of residuals to confirm linearity and homoscedasticity. Statistical significance was set at *p* < 0.05 for all analyses.

### 2.5 An intra-examiner reliability test

An Intraclass Correlation Coefficient (ICC) was calculated for 51 participants. The two readings were taken two days apart. All intra-examiner reliability values are greater than 0.90, indicating excellent intra-examiner reliability for all measurements, with very high consistency between the first and second readings (Table 1).

**Table 1:** An intra-examiner reliability test for the mean maximum mouth opening and the three readings.

Measurement	ICC	Interpretation
Mean MMO	0.9998	Excellent reliability
First Attempt MMO	0.9989	Excellent reliability
Second Attempt MMO	0.9998	Excellent reliability
Third Attempt MMO	0.9999	Excellent reliability

## 3 Results

A total of 400 participants were included in the study. Participants ranged in age from 18 to 70 years, with a mean age of 33 ± 12 years. The sample consisted of 152 males (38%) and 248 females (62%). The overall mean maximum mouth opening (MMMO) was 45.7 ± 6.9 mm (median = 46.3 mm; range: 28.0–66.3 mm). Among age groups, the lowest mean MMMO was observed among participants aged 30–39 years (44.8 ± 7.3 mm), whereas the highest was noted in those older than 60 years (47.8 ± 7.8 mm). A statistically significant difference was found between genders using ANOVA, with males demonstrating greater MMMO than females (47.9 ± 6.7 mm vs. 44.3 ± 6.7 mm; *p* < 0.0001). ANOVA demonstrated significant differences in MMO across height groups (*p* < 0.0001). Tukey *post hoc* testing showed that individuals 171–180 cm tall had significantly greater MMO (49.8 mm) than those ≤150 cm, 151–160 cm, and 161–170 cm. Participants ≤150 cm had the smallest MMO (42.0 mm). No significant differences were found between the >180 cm group and intermediate height groups. These results confirm a strong positive relationship between height and MMO. No statistically significant association was observed between BMI categories and MMMO, although the obese group had the highest mean value (45.8 ± 6.6 mm) (Table 2).

**Table 2:** Mean maximum mouth opening among the participants (mm).

Sociodemographic characteristics	*N*	%	Maximum mouth opening				*p*-value^a^
			Mean	Median	Range	Sd	
Overall	400	100	45.7	46.3	28.0–66.3	6.9	
Age groups (years)							
18–29	179	45	46.5	46.7	29.9–66.3	6.9	0.3023
30–39	109	27	44.8	46.4	27.9–61.6	7.3	
40–49	65	16	45.2	45.1	33.0–61.3	6.9	
50–59	41	10	45.5	46.9	34.5–58.2	5.9	
>60	6	2	47.8	46.7	37.7–60.2	7.8	
Gender							
Male	152	38	47.9	48.1	31.9–66.3	6.7	<0.0001
Female	248	62	44.3	44.3	28.0–62.5	6.7	
Weight							
40–60 kg	132	33	44.8	44.7	28.1–62.5	7.0	0.1113
61–80 kg	174	43	45.6	46.3	28.0–66.3	6.7	
81–100 kg	70	18	47.6	47.9	31.9–61.6	7.0	
101–120 kg	19	5	46.0	46.9	34.1–58.2	7.8	
>20 kg	5	1	43.6	41.1	38.9–49.5	5.1	
Height							
≤150 cm	27	7	42.0	42.9	29.5–57.9	6.6	<0.0001
151–160 cm	171	43	44.3	44.3	28.0–62.5	6.9	
161–170 cm	118	30	46.1	46.7	31.9–60.2	6.2	
171–180 cm	72	18	49.8	48.8	33.9–66.3	6.5	
>180 CM	12	3	46.6	46.5	36.4–58.8	7.2	
BMI							
Underweight	26	7	47.1	47.5	35.5–58.4	6.8	0.7027
Normal weight	167	42	45.6	46.3	28.1–66.3	7.0	
Overweight	109	27	45.4	46.0	28.0–61.3	7.3	
Obese	98	24	45.8	46.3	30.4–61.6	6.6	

a One-way Analysis of Variance (ANOVA).

Table 3 summarizes the anthropometric characteristics of the participants by gender. Males exhibited higher mean height (172.0 ± 6.7 cm) and weight (78.8 ± 18.2 kg) compared with females (157.6 ± 5.6 cm and 65.5 ± 16.5 kg, respectively). BMI values were similar between genders (26.6 ± 5.8 in males vs. 26.4 ± 6.8 in females).

**Table 3:** Distribution of participants' height, weight, and BMI across different genders.

Sociodemographic characteristics	Variables	Mean	Median	Range	SD
Gender					
Male	Height	172.0	171.0	154–189	6.7
	Weight	78.8	76.0	47–149	18.2
	BMI	26.6	26.2	16–44	5.8
Female	Height	157.6	157.0	138–173	5.6
	Weight	65.5	63.0	44–155	16.5
	BMI	26.4	25.2	16.1–70.8	6.8
Overall	Height	163.1	161.0	138–189	9.2
	Weight	70.5	68.5	44–155	18.3
	BMI	26.5	25.4	16.1–70.8	6.4

• Height in cm.

• Weight in kg.

Table 4 presents the distribution of maximum mouth opening categories across sociodemographic and anthropometric variables. Chi-square testing supported by Monte Carlo simulation was used to assess statistical significance. No significant association was found between MMO and age group (*p* = 0.6377). Gender was significantly associated with MMO (*p* = 0.0020), with males more frequently represented in the >50 mm category. Height was also significantly associated with MMO (*p* = 0.0017), with taller individuals more commonly classified within the wider MMO categories. No significant associations were observed between MMO and weight (*p* = 0.3977) or BMI (*p* = 0.7986).

**Table 4:** Maximum mouth opening and participants' demographic and anthropometric variables .

Sociodemographic characteristics	Maximum mouth opening				Total	*P*-Value^a^
	<35 mm	35–39 mm	40–50 mm	>50 mm		
Age (Years)						
18–29	8	24	94	53	179	0.6377
30–39	12	16	54	27	109	
40 49	4	14	32	15	65	
50–59	2	7	24	8	41	
>60	0	1	3	2	6	
Gender						
Male	4	18	77	53	152	0.0020
Female	22	44	130	52	248	
Weight						
40–60 kg	10	22	69	31	132	0.3977
61–80 kg	12	26	95	41	174	
81–100 kg	2	9	32	27	70	
101–120 kg	2	3	8	6	19	
> 120 kg	0	2	3	0	5	
Height						
≤150 cm	4	7	13	3	27	0.0017
151–160 cm	17	28	88	38	171	
161–170 cm	4	20	66	28	118	
171–180 cm	1	4	34	33	72	
>180 cm	0	3	6	3	12	
BMI						
Underweight	0	5	13	8	26	0.7986
Normal weight	11	25	89	42	167	
Overweight	10	16	58	25	109	
Obese	5	16	47	30	98	

a Chi-squared test and Monta Carol simulation test.

A multivariable linear regression model was conducted to identify independent predictors of maximum mouth opening (MMO). The model, which included age, gender, height, and weight, was statistically significant (*p* < 0.0001) and explained 9% of the variance in MMO (*R*^2^ = 0.090). Height was the only variable that remained significantly associated with MMO (*β* = 0.154, *p* = 0.008), indicating that greater stature is independently associated with greater mouth-opening capacity. Gender showed a non-significant trend toward lower MMO among females (*β* = −1.59, *p* = 0.135), while age (*β* = −0.043, *p* = 0.163) and weight (*β* = −0.002, *p* = 0.940) were not significant predictors. These findings suggest that, although several demographic and anthropometric variables vary across individuals, height is the strongest independent determinant of MMO after adjustment for potential confounders.

## 4 Discussion

This study assessed maximum mouth opening (MMO) in a sample of 400 participants and explored its association with sociodemographic and anthropometric variables. The mean MMO observed in our cohort was 45.7 ± 6.9 mm, which lies within the normal physiological range of 50–60 mm reported by Posselt (11). Our findings are consistent with previous Saudi data; El-Abdin et al. (12) measured MMO in 1,158 patients aged 5–70 years and reported a mean of 46.12 mm, whereas Al-Dlaigan et al. examined Saudi adolescents and observed mean MMO values of 43.5 ± 4.23 mm in males and 35.5 ± 4.4 mm in females, confirming a marked sex-related difference (7, 8). International studies have reported similar values: a French population study found an overall mean of 50.77 mm with significant associations with height and age (5), while an Irish cohort reported lower values, with a mean MMO of 43 mm for males and 41 mm for females (13). Studies in the United States have also reported comparable findings in children, with a mean MMO of 43.99 mm in participants aged 4–14 years (14). A study from the University of Gothenburg found mean MMO values of 44.8 ± 9.4 mm for men and 39.2 ± 10.8 mm for women (15). A Scandinavian report indicated values of 58.6 mm in healthy 29-year-old men and 53.3 mm in women, with a wide range of 34–77 mm and 42–75 mm, respectively (4). Even in older adults, MMO has been shown to remain relatively high; one study reported mean values of 52.7 mm for men and 49.3 mm for women aged 70 years with residual dentition (16). The convergence of these findings across populations supports the generalizability of our results and highlights that gender consistently emerges as a strong determinant of MMO.

### 4.1 Sex differences in maximum mouth opening

In the present study, males demonstrated a mean MMO of 47.9 ± 6.7 mm compared with 44.3 ± 6.7 mm in females, a difference of 3.6 mm that reached statistical significance (*p* = 0.0020). This difference is in line with other reports that consistently document a sex-related gap of 3–5 mm (5, 15). The difference is commonly attributed to greater mandibular dimensions and muscle mass in males, which may contribute to wider oral opening. Clinically, this emphasizes the importance of sex-specific reference values when assessing patients for trismus, commonly defined as MMO < 35 mm. Using a single universal cutoff may risk underdiagnosis in males and overdiagnosis in females.

### 4.2 Association between body height, BMI, and MMO

Height was also significantly associated with MMO (*p* = 0.0017), with participants ≥171 cm tall exhibiting MMO values nearly 5 mm greater than those ≤150 cm. This positive association has been reported previously, with several studies demonstrating that craniofacial dimensions scale with body size, leading to larger oral openings in taller individuals (1, 15). This observation is of practical importance for clinicians, as expected, MMO should be interpreted relative to the patient's stature. In contrast, BMI did not show a significant association with MMO in this study (*p* = 0.7986), despite underweight participants having slightly higher mean MMO 47.1 mm compared with overweight individuals 45.4 mm. While some reports have suggested that weight and BMI may influence MMO (5), our findings support the view that obesity does not inherently limit mandibular mobility, which is reassuring for clinical procedures that require adequate oral access, including prosthodontic impressions, surgical extractions, and intubation.

### 4.3 Age-related variations in MMO

Interestingly, no significant association was observed between MMO and age (*p* = 0.6377), although participants aged >60 years demonstrated the highest mean MMO (47.8 mm), whereas those aged 30–39 years had the lowest (44.8 mm). This finding contrasts with studies reporting progressive decline of MMO with advancing age due to temporomandibular joint remodeling, muscle atrophy, and changes in dentition (13, 16). The relatively higher MMO observed among our oldest participants may reflect selection bias, as only healthy and largely dentate individuals were included, or could indicate preserved neuromuscular adaptation in this population.

### 4.4 Measurement reliability and reproducibility

The present study demonstrated excellent consistency between the averaged maximum mouth opening and the three repeated measurements, with correlation coefficients exceeding 99.8%. Notably, the first attempt exhibited the smallest deviation from the averaged value, with a mean absolute percentage difference of only 0.25%, compared with 0.62% and 0.72% for the second and third attempts, respectively. This finding suggests that the initial measurement may provide the most accurate and clinically representative estimate of maximum mouth opening. Comparable trends have been reported in studies assessing mandibular movement reproducibility, where the first attempt often shows superior concordance with reference values before the influence of participant fatigue or neuromuscular adaptation becomes apparent (2, 4, 7). These results reinforce the validity of the measurement protocol and highlight that a single well-standardized attempt may be sufficient for clinical screening and epidemiological studies, while repeated trials remain essential in research requiring maximal precision.

### 4.5 Clinical implications and recommendations

Collectively, these findings reinforce the need for population-specific and demographic-specific reference values for MMO to improve diagnostic accuracy for restricted jaw opening. The observation that the average MMO is approximately 48 mm for males and 44 mm for females can guide clinicians in defining normal ranges and setting realistic expectations for oral procedures. Moreover, the lack of association between BMI and MMO suggests that body weight should not be relied upon as a predictor of mandibular hypomobility. Our results are strengthened by the large sample size and standardized measurement protocol.

### 4.6 Limitations

This study has several limitations that merit consideration. First, the cross-sectional design limits the ability to establish causal or temporal relationships between sociodemographic or anthropometric characteristics and maximum mouth opening (MMO). Second, although the overall sample size was adequate, the reliance on non-probability sampling may introduce selection bias and restrict the generalizability of the findings to the broader Saudi population. Third, MMO was measured during a single examination session, which may not fully account for natural intra-individual variability arising from diurnal fluctuations, neuromuscular fatigue, or transient temporomandibular stiffness. Fourth, although participants with overt temporomandibular symptoms were excluded, no comprehensive clinical or radiographic assessment was performed, and subclinical temporomandibular joint alterations may therefore have gone undetected. Finally, the sample consisted of individuals who were sufficiently healthy and willing to participate, raising the possibility of a healthy volunteer effect, which may partly explain the relatively preserved MMO observed among older adults. Despite these limitations, the large sample size, standardized measurement procedure, and excellent intra-session reliability support the robustness of the findings and their relevance for clinical assessment and population-based reference standards.

## 5 Conclusion

In this study, the mean maximum mouth opening (MMO) was 45.7 ± 6.9 mm, with significantly greater values observed in males and taller participants, whereas age, weight, and BMI were not associated with MMO. Height remained the strongest independent predictor of mandibular opening capacity in multivariable analysis. These findings support the use of sex and stature-adjusted reference values to enhance the accuracy of trismus diagnosis and inform clinical decision-making in oral and maxillofacial practice. The results highlight the need for population specific normative standards rather than universal thresholds. Future studies incorporating longitudinal follow-up and craniofacial morphology measurements are warranted to refine diagnostic criteria and improve clinical assessment of mandibular mobility.

## Data Availability

The raw data supporting the conclusions of this article will be made available by the authors, without undue reservation.
